# Association between intrapleural urokinase monotherapy and treatment failure in patients with pleural infection: a retrospective cohort study

**DOI:** 10.1186/s12890-023-02559-5

**Published:** 2023-07-21

**Authors:** Jumpei Taniguchi, Hiroki Matsui, Tatsuya Nagai, Ayumu Otsuki, Hiroyuki Ito, Hiroshi Sugimura, Kei Nakashima

**Affiliations:** 1 Department of Pulmonology, Kameda Medical Center, 929 Higashi-cho, Kamogawa, 296- 8602 Chiba Japan; 2 Department of Clinical Epidemiology and Health Economics, School of Public Health, The University of Tokyo, Tokyo, Japan; 3 Clinical Research Support Office, Kameda Medical Center, Chiba, Japan; 4 Department of Thoracic Surgery, Kameda Medical Center, Chiba, Japan

**Keywords:** Drainage, Empyema, Retrospective study, Pleural infection, Urokinase

## Abstract

**Background:**

Pleural infection, an infection of the pleural space, is frequently treated with antibiotics and thoracic tube drainage. In case of insufficient drainage, an intrapleural fibrinolytic agent is considered before surgical intervention. However, the effectiveness of fibrinolytic monotherapy is still controversial. Therefore, we aimed to examine the association between urokinase monotherapy and treatment failure in patients with pleural infection.

**Methods:**

In this retrospective observational study, patients with pleural infection underwent chest tube insertion were divided into two groups including patients treated with or without intrapleural instillation of urokinase. The propensity score overlap weighting was used to balance the baseline characteristics between the groups. Treatment failure was defined by the composite primary outcome of in-hospital death and referral for surgery.

**Results:**

Among the 94 patients, 67 and 27 patients were in the urokinase and non-urokinase groups, respectively. Urokinase monotherapy improved the composite outcome between the groups (19.4% vs. 48.1%, p = 0.01). After adjusting using propensity score overlap weighting, urokinase monotherapy improved the composite outcome compared to the non-urokinase group (19.0% vs. 59.5%, p = 0.003).

**Conclusions:**

Urokinase monotherapy can be an important nonsurgical treatment option for patients with pleural infection.

**Trial registration:**

The participants were retrospectively registered.

**Supplementary Information:**

The online version contains supplementary material available at 10.1186/s12890-023-02559-5.

## Background

Pleural infection is an infection of the pleural space, which occurs following pneumonia or pneumonia-associated pleural effusion [1]. Pleural infection affects more than 65,000 patients annually in the United Kingdom and the United States alone, with 12-month mortality rates ranging from 10 to 20%, as well as a gradual increase in incidence [2, 3]. Treatment of pleural infection consists of appropriate antibiotic therapy along with adequate drainage of the infected pleural fluid [4]. Percutaneous catheter drainage is the primary intervention to reduce infected pleural effusion. However, it is not always successful because of the presence of thick fibrin layer and multifocal and localized effusions [5]. In such cases, surgical procedures must be considered. However, it may be difficult to perform surgical treatment in cases with poor access to surgical intervention, high surgical risk owing to the advanced age or poor general health condition, or refusal of surgical intervention. Intrapleural fibrinolysis is a widely accepted therapy in which plasminogen converted to plasmin is catalyzed followed by the degradation of fibrin-to-fibrin degradation products, which helps to break up clots and reduce the viscosity of thick, sticky material thereby promoting drainage of the pleural cavity [6].

Urokinase, also known as urokinase-type plasminogen activator, is one of the most widely used fibrinolytic agents [7]. Previous small-scale randomized controlled trials (RCTs) and combined systematic reviews and meta-analyses have suggested that it might contribute to reducing the rate of surgical referrals and improving clinical and radiological outcomes [8–12]. However, heterogeneity of patients and treatment plans in these studies and potential biases have led to inconclusive results. In addition, in 2005, the largest RCT of fibrinolytic monotherapy using streptokinase, which is considered to have a similar mechanism to urokinase, failed to show efficacy in patients with pleural infection [6]. Therefore, consensus guidelines do not recommend routine use of fibrinolytic agents for complicated pleural effusions and early empyema [1, 12]. In 2011, the largest RCT was conducted on fibrinolytics, in which 210 patients with empyema were randomly assigned to one of four intrapleural treatment groups. The authors demonstrated that dual therapy combining tissue plasminogen activator(tPA) and deoxyribonuclease (DNase) resulted in a significant reduction in surgical referrals [13]. Consequently, dual therapy has replaced fibrinolytic monotherapy as the standard treatment. However, concerns have been raised regarding the use of fibrinolytics and DNase combined and the potential increased risk of serious haemorrhagic side effects, such as pleural haemorrhage and haemoptysis, compared with the use of fibrinolytics alone [13, 14]. Moreover, owing to the high cost and limited availability of dual therapy involving specific fibrinolytic agents such as tPA and DNase, fibrinolytic monotherapy such as urokinase remains a widely used treatment option, particularly in settings with limited resources [15, 16]. However, data from studies revealing the association between fibrinolytic monotherapy and treatment failure are still insufficient.

In this study, we retrospectively evaluated the effectiveness of urokinase monotherapy in patients with pleural infection to determine whether urokinase treatment resulted in a difference concerning treatment failure of pleural infection.

## Methods

### Study population

To evaluate whether intrapleural urokinase reduces treatment failure, this study involved patients with stage II or early-stage III acute pleural infection, for whom there is limited evidence regarding the use of intrapleural urokinase [1].

The basic method of patient enrolment and collection of variables in this study was consistent with that in a previous study [17]. We retrospectively collected data from adult patients (≥ 18 years) with chest tube placement for pleural infection who were admitted to the 917-bed Kameda Medical Center, Japan between January 2011 and July 2021. The inclusion criteria were as follows: (1) hospitalized patients; (2) patients diagnosed with pyothorax without fistula (J869) according to the International Classification of Diseases, 10th revision (ICD-10) on admission (cases in which the physician diagnosed stage II–III pleural infection based on the characteristics of imaging studies and pleural effusion); and (3) patients who underwent continuous chest tube insertion or percutaneous pleural effusion drainage during hospitalization (coded as J019, K496-5 in the Japanese medical service fee points). The exclusion criteria included patients: (1) with traumatic pleural infection; (2) with malignant pleural effusion; (3) with chronic empyema (highly organized pleural effusion and/or fibrinous pleural covering); and (4) who were referred to a surgeon for surgery before or immediately after chest tube placement.

### Ethics approval and consent to participate

This retrospective cohort study was reviewed and approved by the Research Ethics Committee of Kameda Medical Center (#21–091) in accordance with the Declaration of Helsinki of 1964 and all its subsequent amendments. The Ethics Committee of Kameda Medical Center waived the requirement for written informed consent owing to the retrospective nature of the study; participants were also given the option to opt-out.

### Management of patients in this study

We initiated empiric intravenous antibiotic therapy of all patients diagnosed with pleural infection. After identification of causative bacteria, the antibiotics were changed based on the drug susceptibility test findings.

Chest tubes were selected ranging from 12Fr to 32Fr at the discretion of the attending physician and inserted under ultrasound or X-ray/computed tomography (CT) guidance. A traditional three-chamber plastic unit was used as the chest drainage system. After insertion of the chest tube, a water suction level of 0 to − 20 cm was used depending on the drainage volume.

### Urokinase use

The exposure in this study was the use of urokinase. Intrapleural urokinase could be used for patients with inadequate drainage after antibiotics and drainage therapy at the discretion of the attending physician. Given the unclear efficacy of urokinase monotherapy in treating pleural infections, some physicians favoured only standard care comprising antibiotics and chest tubes rather than urokinase monotherapy. When urokinase was used, it was administered at a dose of 120,000 units was administered once daily for a period determined by the attending physician. In this study, only urokinase was used as a fibrinolytic agent, as it was the only one available in our hospital. Other fibrinolytic agents such as tPA, streptokinase, and DNase were not used in this study.

The patients showing antibiotic and appropriate drainage (± urokinase) treatment failure, associated outcomes including persistent or worsening pleural effusion, new fever, leucocytosis, and elevated inflammatory markers, were referred for surgery.

### Outcome

The outcome was treatment failure, which was defined as a composite outcome of hospital death and referral for surgery.

### Data collection and RAPID score

In this retrospective cohort study, we collected demographic and clinical data for patients including age, sex, body mass index (BMI), laboratory results at admission, pleural fluid analysis at thoracentesis or chest tube placement, and imaging data on the patient population and compared them according to the exposure. This data included the RAPID score, a validated prognostic score in patients with pleural infection [18]. The score may help to risk-stratify patients with pleural infection based on five characteristics [renal failure (urea), age, fluid purulence, infection source (hospital vs. community), and dietary factors (albumin)] [RAPID] [19]. We calculated the RAPID score corresponding to the parameters shown in Supplementary Tables 1, enabling us to identify those at low risk [score 0–2], medium risk [score 3–4], and high risk [score 5–7] of mortality from a pleural infection [20].

### Statistical analyses

Continuous variables were analysed using the Wilcoxon rank-sum test, while categorical variables were analysed using the chi-square test. We adjusted for patient background using propensity score overlap weighting and estimated the treatment effect of exposure [21, 22]. We calculated the propensity score for exposure using a logistic regression method adjusting for confounders (age, sex, BMI, C-reactive protein [CRP], pleural fluid characteristics [culture positive for bacteria, pH, and glucose], X-ray/CT-guided chest tube insertion, and RAPID score) as predicting variables [19, 23–25]. After weighting, we measured differences between each group using standardized mean differences (SMD) for the covariates. An SMD lower than 0.1 indicated a good covariate balance [26]. Statistical analyses were performed by the R software (version 3.6.3; R Development Core Team, https://www.r-project.org/).

## Results

**Fig. 1:**
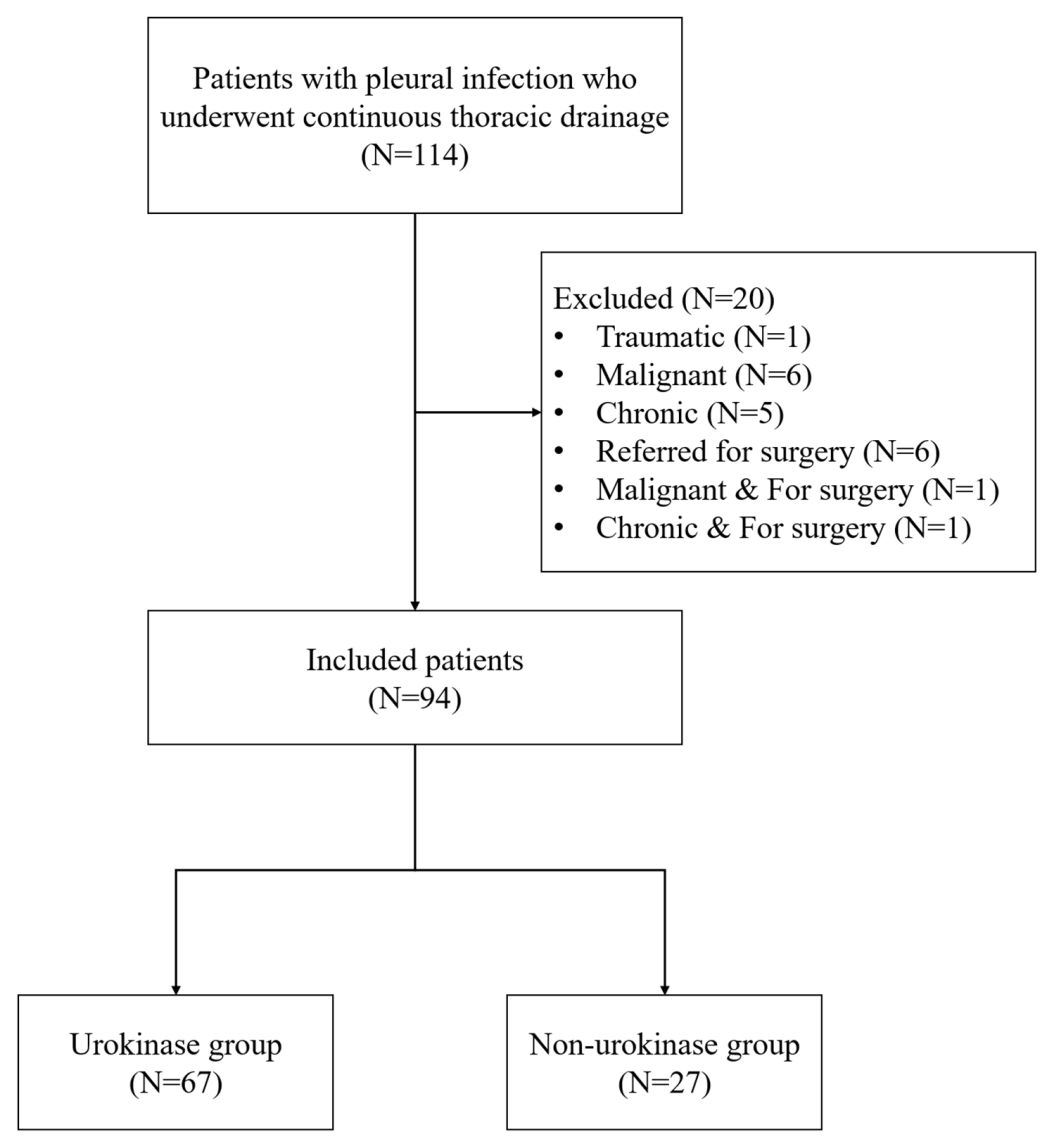
Flowchart for selection of patients

The patient selection process is outlined in Fig. 1. A total of 114 patients were initially included in the study, of which 20 were excluded based on the exclusion criteria shown in Fig. 1. The final study sample consisted of 94 patients.

Table 1 presents the unweighted and weighted demographic and clinical characteristics of the eligible patients, stratified by whether or not they received urokinase therapy. In unweighted patient characteristics, the urokinase group had a slightly lower median age of 69.0 years (interquartile range [IQR]: 61.5–76.5) than that of the non-urokinase group (median: 75.0 years; IQR: 65.5–81.0), however, there was no significant difference between the two groups. Moreover, there were no significant differences in sex, BMI, and CRP level. The median pleural fluid pH, glucose, and LDH levels were 7.34 (IQR: 7.21–7.50), 19.0 mg/dL (IQR: 1.0–71.3), and 1343.5 U/L (IQR: 835.3–2432.3), respectively, in the urokinase group, and 7.41 (IQR: 7.13–7.53), 33.0 mg/dL (IQR: 1.0–107.5), and 1665.0 U/L (IQR: 560.5–12090.0), respectively, in the non-urokinase group; no significant differences were observed between the two groups. The X-ray/CT-guided chest tube insertion and chest tube size were decided by the physician, but there were no related significant differences between the two groups. The RAPID scores were also not significantly different between the two groups. In the urokinase group, the urokinase treatment period was 3.5 ± 1.43 days (mean ± standard difference).

**Table 1:** Patient characteristics classified by urokinase in unweighted and weighted study populations

	Unweighted study population				Weighted study population				
	Urokinase group (n = 67)	Non-urokinase group (n = 27)	p value	SMD	Urokinase group (n = 13.54)	Non-urokinase group (n = 13.54)	p value	SMD	
Age, years (Median ± IQR)	69.0 (61.5–76.5)	75.0 (65.5–81.0)	0.139	0.178	70.3 (63.7–78.0)	71.0 (64.0–80.5)	0.700	< 0.001	
Female, n (%)	9 (13.4)	4 (14.8)	1.000	0.040	1.5 (10.8)	1.5 (10.8)	1.000	< 0.001	
BMI, kg/m^2^ (Median ± IQR)	20.2 (17.9–24.4)	20.3 (17.7–24.7)	0.944	0.034	21.2 (18.3–25.0)	22.2 (17.7–25.3)	0.988	< 0.001	
CRP, mg/dL (Median ± IQR)	18.7 (11.8–27.8)	17.5 (11.9–21.7)	0.240	0.334	18.6 (8.9–26.1)	19.4 (13.0–23.8)	0.954	< 0.001	
Pleural-fluid characteristic									
Culture positive for bacteria, n (%)	28 (41.8)	18 (66.7)	0.051	0.516	8.8 (64.6)	8.8 (64.6)	1.000	< 0.001	
pH, (Median ± IQR)	7.34 (7.21–7.50)	7.41 (7.13–7.53)	0.951	0.162	7.29 (7.13–7.45)	7.28 (7.12–7.49)	0.873	< 0.001	
Glucose, mg/dL (Median ± IQR)	19.0 (1.0–71.3)	33.0 (1.0–107.5)	0.546	0.319	14.5 (1.0–70.7)	6.45 (0.0–69.4)	0.748	< 0.001	
LDH, IU/mL (Median ± IQR)	1343.5 (835.3–2432.3)	1665.0 (560.5–12090.0)	0.652	0.467	1452.3 (640.8–2898.0)	1462.1 (537.7–15154.8)	0.748	0.322	
X-ray/CT-guided chest tube insertion, n (%)	13 (19.4)	6 (22.2)	0.981	0.069	2.9 (21.4)	2.9 (21.3)	0.994	0.002	
Chest tube size, French			0.310	0.360			1.000	< 0.001	
≤ 14, n (%)	13 (19.4)	8 (29.6)			3.2 (24.0)	3.2 (24.0)			
15–20, n (%)	32 (47.8)	14 (51.9)			7.0 (51.9)	7.0 (51.9)			
> 20, n (%)	22 (32.8)	5 (18.5)			3.3 (24.1)	3.3 (24.1)			
RAPID score			0.127	0.454			1.000	< 0.001	
Low-risk, n (%)	18 (26.9)	8 (29.6)			3.7 (27.2)	3.7 (27.2)			
Medium-risk, n (%)	40 (59.7)	11 (40.7)			5.8 (42.7)	5.8 (42.7)			
High-risk, n (%)	9 (13.4)	8 (29.6)			4.1 (30.2)	4.1 (30.2)			

BMI: body mass index; CT: computed tomography; CRP: C-reactive protein; IQR: interquartile range; LDH: lactate dehydrogenase; RAPID: renal, age, fluid purulence, infection source, and dietary factors; SMD: standardized mean difference

After adjusting using propensity score overlap weighting, the SMDs for age, sex, BMI, CRP, X-ray/CT-guided chest tube insertion, pleural fluid characteristics (culture positive for bacteria, pH, and glucose), chest tube size, and the RAPID score were < 10%; all confounders were well-balanced.

Clinical outcomes stratified according to the urokinase use are shown in Table 2. The unweighted composite outcome was 13 (19.4%) in the urokinase group and 13 (48.1%) in the non-urokinase group, with a significant difference between the two groups (p = 0.01). The number of deaths reported was two in the urokinase group and four in the non-urokinase group; 11 (16.4%) and 9 (33.3%) patients, respectively, underwent surgical procedures. However, each individual outcome (death during hospitalization and referral for surgery) did not differ significantly between the two groups. In the weighted study population, the composite outcome and referral for surgery were 2.6 (19.0%) and 1.5 (11.0%) in the urokinase group compared to 8.1 (59.5%) and 5.7 (41.9%), respectively, in the non-urokinase group, with a significant difference in improvement in both composite outcome and surgical intervention. However, there was no significant difference in death during hospitalization between the two groups. The length of hospital stay and the time from drain insertion to surgery are shown in Supplementary Table 2. The median hospital stay was 21.0 (IQR: 17.0, 30.5) and 24.0 (IQR: 15.0, 43.0) days for the urokinase and non-urokinase groups, respectively, with no statistically significant difference observed between groups (p = 0.569). The median time to surgery from drain insertion was 14.0 (IQR: 8.0, 16.0) and 13.0 (IQR: 8.0, 14.0) days in the urokinase and non-urokinase groups, respectively, with also no statistically significant difference observed between groups (p = 0.549).

**Table 2:** Clinical outcomes classified by urokinase in unweighted and weighted study populations

	Unweighted study population				Weighted study population			
	Urokinase group (n = 67)	Non-urokinase group (n = 27)	p value	SMD	Urokinase group (n = 13.5)	Non-urokinase group (n = 13.5)	p value	SMD
Composite outcome^†^, n (%)	13 (19.4)	13 (48.1)	0.010	0.638	2.6 (19.0)	8.1 (59.5)	0.003	0.912
Death during hospitalization, n (%)	2 (3.0)	4 (14.8)	0.098	0.425	1.1 (8.0)	2.4 (17.6)	0.367	0.292
Referral for surgery, n (%)	11 (16.4)	9 (33.3)	0.125	0.399	1.5 (11.0)	5.7 (41.9)	0.006	0.747

^†^Composite outcome: death during hospitalization and referral for surgery

SMD: standardized mean difference

There were no serious adverse events, such as allergic reactions to urokinase, haemoptysis, pleural haemorrhage involving hemodynamic change, decrease in haemoglobin level, and need of surgery, in any case during the study period.

## Discussion

In this study, we retrospectively evaluated the association between urokinase monotherapy and treatment failure in patients with pleural infection. Our study comprising 94 patients with pleural infection showed improvement in the composite outcome of referral to a surgeon and death during hospitalization. The findings indicated that urokinase monotherapy may still be an important treatment option for pleural infection in hospitalized patients belonging to regions or countries with poor surgical resources or patients with high surgical risk. Our result of improvement in the composite outcome, death during hospitalization and surgical referral, was consistent with the results of several previous RCTs and integrated systematic reviews on urokinase [9, 10, 12]. Further, our findings have two important clinical strengths compared to previously reported studies.

First, compared with previous RCTs, we included patients of older age. Urokinase monotherapy for patients with pleural infection was well studied in the 2000s. However, previous RCTs included relatively young patients, with a median age of approximately 50 years [8, 9, 11]. In our study, we included older patients, with a median age approximately 70 years, having a high risk for surgery. To the best of our knowledge, the effectiveness of urokinase in elderly population is still not well understood, and this study is meaningful and will contribute to the body of knowledge.

Second, in this a retrospective observational study, we adjusted for patient background using propensity score overlap weighting for many confounding factors, including the RAPID score, which is a prognostic predictor [18]. Since the 2001 RCT, observational studies on urokinase for patients with pleural infection have switched to different doses or combination therapy such as ozone or saline flushing or DNase [27–30]. However, pleural infection and its treatment method are highly heterogeneous in nature, and confounding factors might be problematic in retrospective studies. Our results could have higher internal validity than those of previous placebo-based retrospective observational studies because we adjusted for confounding factors, including the RAPID score.

There is a continued debate concerning the optimal intrapleural fibrinolytic treatment for pleural infection. Early RCTs showed that the use of intrapleural fibrinolytic agents in addition to chest tube drainage led to a notable reduction in surgical interventions [9, 10]. In contrast, a large multicentre RCT in the UK using streptokinase did not show clinical benefits of intrapleural fibrinolytic monotherapy [6]. Conflicting results of other studies in different settings prompted multiple reviews [12, 31, 32]. The most recent Cochrane review remains inconclusive; however, it suggests that fibrinolytic monotherapy, particularly urokinase monotherapy, may not improve mortality but reduce surgical intervention [12].

Since the efficacy of the dual therapy of fibrinolytic agents and DNase has been demonstrated, the combination has been the mainstay of intrapleural fibrinolytic therapy [13]. However, there are some concerns about side effects, such as pleural haemorrhage and haemoptysis, of the dual therapy [13]. Therefore, its use should be considered on a case-by-case basis for patients with a high risk of bleeding [33]. It has been suggested that urokinase monotherapy is less likely to cause bleeding or allergic side effects than other fibrinolytic agents [11, 12]. Similarly, there were no incidences of allergic reactions, pleural haemorrhage, haemoptysis, or other serious side effects in our study. Urokinase monotherapy, unlike other fibrinolytic monotherapy, might have the potential to improve clinical outcomes of death during hospitalization and surgical referrals without any increase in serious side effects. However, since the predominance of small-scale and old RCTs with high or unknown risk of bias, small-scale retrospective observational studies with heterogeneity of patients and treatment patterns, large-scale RCTs with low risk of bias, or large observational studies adjusted for many confounding factors are needed in the future to conclude the association between urokinase monotherapy and treatment failure.

Our study has some limitations. First, being a retrospective, single-centre study, it lacks a standardized protocol; the choice of antibiotics and techniques, type and size of drains used, type of medical professions performing thoracic drainage, choice of subsequent treatment in case of treatment failure, and use of urokinase and its duration of use were adapted as per the individual needs of each patient and judgement of the treating physician. Unmeasured confounding factors could be problematic, especially since the final decision to use urokinase was made at the discretion of the each attending physician, which may make it difficult to extrapolate our conclusions to other facilities.

Second, the sample size was small. After adjusting for many confounding factors, the weighted study population was 13.5. Although our study showed that urokinase significantly improved the composite outcome, inadequate sample size can be a threat to internal and external validity of our results. Ideally, a large-scale RCT or large-scale observational study adjusted for many confounding factors is needed to confirm our result.

## Conclusions

In this study, urokinase monotherapy in patients with pleural infection resulted in improvement of the composite outcome comprising outcomes of death during hospitalization and referral for surgery. Urokinase monotherapy can be an important treatment option for patients with pleural infection, especially in cases with poor access to surgical intervention, high surgical risk owing to the advanced age or poor general health condition. However, further large-scale RCTs or large-scale observational studies are needed to confirm our result.

## Electronic supplementary material

Below is the link to the electronic supplementary material.

Supplementary Material 1

Supplementary Material 2

## Data Availability

The data supporting the findings of this study are available from the corresponding author upon reasonable request. The data were not publicly available because of privacy and ethical restrictions.

## Abbreviations

BMI: body mass index
CRP: C-reactive protein
DNase: deoxyribonuclease
ICD: International Classification of Diseases
IQR: interquartile range
LDH: lactate dehydrogenase
RAPID: renal, age, fluid purulence, infection source, and dietary factors
RCT: randomized controlled trial
SMD: standardized mean difference
tPA: tissue plasminogen activator
