# Supplementary material for: Association between intrapleural urokinase monotherapy and treatment failure in patients with pleural infection: a retrospective cohort study
Source: BMC Pulm Med. 2023 Jul 21;23:273. doi: 10.1186/s12890-023-02559-5 (PMC10362621; doi:10.1186/s12890-023-02559-5)
Supplement: Supplementary file 2 — Supplementary Material 2 [file 12890_2023_2559_MOESM2_ESM.docx]

Supplementary Table 2. Length of hospital stay and time to surgery from drain insertion classified by urokinase

|  | Urokinase group  (n = 67) | Non-urokinase group  (n = 27) | p value |
| --- | --- | --- | --- |
| Length of hospital stay, days  (Median ± IQR) | 21.0 (17.0-30.5) | 24.0 (15.0-43.0) | 0.569 |
| Time to surgery from drain insertion, days (Median ± IQR) | 14.0 (8.0-16.0) | 13.0 (8.0-14.0) | 0.549 |

IQR: interquartile range
